# Distribution of fungi and their toxic metabolites in melon and sesame seeds marketed in two major producing states in Nigeria

**DOI:** 10.1007/s12550-020-00400-0

**Published:** 2020-07-14

**Authors:** Adetoun O. Esan, Stephen O. Fapohunda, Chibundu N. Ezekiel, Michael Sulyok, Rudolf Krska

**Affiliations:** 1 Department of Microbiology, Babcock University, Ilishan Remo, Ogun State Nigeria; 2 Department of Agrobiotechnology (IFA–Tulln), Institute of Bioanalytics and Agro–Metabolomics, University of Natural Resources and Life Sciences Vienna (BOKU), Konrad Lorenzstr. 20, A–3430 Tulln, Austria; 3 Institute for Global Food Security, School of Biological Sciences, Queen’s University Belfast, University Road, Belfast, Northern Ireland BT7 1NN UK

**Keywords:** Food safety, Melon, Mycology, Mycotoxins, Sesame

## Abstract

In this study, melon (*n* = 60) and sesame (*n* = 60) seeds purchased from markets within Benue and Nasarawa states, respectively, in Nigeria, during two seasons (dry and wet), were analysed for fungal and mycotoxin contamination in order to determine the safety of these foods for human consumption. Molecular analysis revealed the following seven fungal taxonomic groups in the foods: *Aspergillus* section *Candidi*, *Aspergillus* section *Flavi*, *Aspergillus* section *Nigri*, *Cladosporium*, *Fusarium fujikuroi* species group, *Penicillium*, and Pleosporales/Didymellaceae. A total of 78 microbial metabolites, including several mycotoxins, occurred in the foods. The most frequent mycotoxins in melon and sesame were aflatoxin B_1_ (occurrence: 76%) and alternariol monomethyl ether (occurrence: 59%), respectively. However, higher mean total aflatoxin levels occurred in sesame (17 μg kg^−1^) than in melon (11 μg kg^−1^). About 28 and 5% of melon and sesame, respectively, exceeded the 4 μg kg^−1^ total aflatoxin limit for oilseeds intended for direct human consumption in the European Union. Additionally, fumonisin B_1_ and moniliformin occurred only in sesame, whilst ochratoxins A and B occurred only in melon; ochratoxin B being reported for the first time in this food. Our data indicated seasonal variations in the fungal and mycotoxin contamination levels in both foods.

**Electronic supplementary material:**

The online version of this article (10.1007/s12550-020-00400-0) contains supplementary material, which is available to authorized users.

## Introduction

Mycotoxin contamination of food resulting from fungal invasion and subsequent biosynthesis of the toxic secondary metabolites is a global challenge, posing a huge hurdle to availability of safe food in regions (e.g. sub-Saharan Africa) where food safety systems are poorly developed (Ezekiel et al. 2019). Poor agricultural practices and substandard postharvest food handling facilities together with climate change, characterized with sporadic fluctuations of temperature, rainfall patterns and drought, have been suggested to raise mycotoxin levels and increase food safety risks in the coming years (Medina et al. 2014; UNEP 2016). Contamination of foods by mycotoxins may affect consumer health, negatively impact trade, and lead to economic decline (IARC 2015; Ezekiel et al. 2019). Consequently, all foods, especially those with dual purposes (staples and prime cash crops) should be considered of merit for mycotoxin control.

Melon (*Colocynthis citrullus* L.) and sesame (*Sesamum indicum*) seeds are widely grown in West and Central African countries, and Nigeria ranks first and fifth, respectively, on the global list of highest producing nations. Specifically, Nigeria contributed 568,940 and 450,000 t to the global production of 925,422 and 5,631,443 t of melon and sesame seeds, respectively, in 2016 (FAOSTAT 2019a). However, the export quantities of melon and sesame seeds from Nigeria in 2016 were 5 and 172,839 t, respectively (FAOSTAT 2019b). One major limiting factor to the export of both crops, especially melon seeds, to the European Union territory (the major market for several African countries) is contamination with mycotoxins (chiefly aflatoxins) beyond the regulatory limits of 2 and 4 μg kg^−1^ for aflatoxin B_1_ and total aflatoxins, respectively, set by the European Commission for oilseeds intended for direct human consumption (European Commission 2010; RASFF 2019). At present, aflatoxins are not regulated in both crops at the local market in Nigeria. Therefore, there is a continuous need for monitoring and tracking compliant and violative agricultural commodity shipments at the local market before they get to the international market.

In Nigeria, melon and sesame are mainly produced as important food and cash crops in the North central parts. Benue and Nasarawa are the major producers of melon and sesame seeds, respectively (NAERLS 2010; Ogbonna and Ejimofor 2013). Both crops are regarded as high energy and oil seeds containing diverse minerals, vitamins and antioxidants (Onyeike and Acheru 2002; Borchani et al. 2010) with additional high protein levels found in melon (Gorskis 1985; Bande et al. 2012). Previous studies have reported the presence of fungal contaminants and/or mycotoxins in melon (Bankole 1993; Bankole et al. 1999, 2004, 2006; Fagbohun et al. 2011; Adeleke et al. 2012; Fapohunda et al. 2014; Williams et al. 2015; Ezekiel et al. 2016; Nwokocha and Opara 2016) and sesame (Mbah and Akueshi 2009; Ezekiel et al. 2012, 2014; Makun et al. 2014; Fapohunda et al. 2012, 2018; Ogara et al. under review) in Nigeria. However, there is no study comparing the fungal and mycotoxin profiles of these crops sampled across two seasons from any of these top-ranked producing states.

In order to protect consumer health and monitor the compliance of food intended for the international market, routine mycotoxin surveillance of melon and sesame seeds available at local markets, within the top-producing states, is required. Thus, this study aimed at determining the fungal profile and spectrum of their toxic metabolites in melon and sesame seeds available in major markets during two seasons (dry and wet) in the highest producing states, Benue and Nasarawa, respectively.

## Materials and methods

### Study design and food sampling

A seasonal mycotoxin surveillance study was conducted within each state ranked as the highest producer of melon and sesame seeds in Nigeria. Thus, Benue state was selected for sampling of melon while Nasarawa state was chosen for sampling of sesame. In each state, markets were selected through a multistage process in order to obtain a good representation of the state. Precisely, the three senatorial districts in each state were selected and two major markets in each district were randomly identified. The senatorial districts (and markets) include Benue South (Otukpo and Tiv), Benue North-East (Dealer and Katsina-ala) and Benue North-West (Markudi ultra-modern and Railway); Nasarawa North (Akwanga and Garaku), Nasarawa South (Doma and Kasunkwaro) and Nasarawa West (Gunduma and Keffi). In each market, five randomly identified vendors of the specified crop were selected. Consequently, 30 vendors were selected for sampling in each state (i.e. 60 vendors for both states).

Each selected vendor had at least 1 t worth of seeds at the time of sampling. Sampling was performed during the following two seasons in 2017: dry season (February and March) and wet season (July). One bulk (~1 kg) sample of the specified crop was collected from each vendor. Altogether, 120 samples were collected as follows: melon (*n* = 60) and sesame (*n* = 60). Equal sample number (*n* = 30) per crop type was collected in each season, such that the total sample size by season was dry season (*n* = 60) and wet season (*n* = 60). Each bulk (~1 kg) sample collected comprised of thoroughly mixed sub-samples obtained from at least five randomly selected bags (50 kg each) per crop lot (1 t). For each of the sampled bags, the sub-samples (~200 g) were drawn from the top, middle and bottom parts using a metal probe. The samples were collected into clean polyethylene bags, labelled and transported immediately to the laboratory for further analysis. At the laboratory, each sample was ground into fine flour in an electric blender (MX-AC400, Panasonic, India), batched into two (A for mycological analysis and B for mycotoxin analysis) and stored at 4 °C (batch A) and − 20 °C (batch B) prior to analysis.

### Mycological analysis of food samples

#### Isolation of moulds

Moulds in all the food samples were recovered by dilution plating according to Samson et al. (1995). Briefly, 10 g of each sample was diluted in 90 mL of sterile distilled water and homogenized for 2 min. Aliquots of 0.1 mL from the homogenized samples were spread-plated in duplicates on freshly prepared potato dextrose agar (PDA; Lab M, UK) supplemented with 30 mg/L chloramphenicol. The inoculated plates were incubated for 3 days at 30 °C in order to mimic the optimum temperature in the region. Thereafter, distinct fungal colonies with different colony ornamentations were purified on freshly prepared ¼ strength PDA plates (9.75 g; 2% agar/L of distilled water). Purified colonies were maintained at 4 °C as malt extract agar (MEA) slants in 4 mL vials.

#### Characterization of moulds

The identification of moulds recovered from the food samples was based on phenotypic and molecular techniques. All purified moulds were preliminarily identified to genus or group level by assessing phenotypic (macro- and micro-) characters of the colonies. Fungi were plated on MEA and incubated at 30 °C for 7 days prior to morphological character assessment. Preliminary assessments were performed in accordance with keys and descriptions in Frisvad and Samson (2004), Leslie and Summerell (2006), Pitt and Hocking (2009), Samson et al. (2011) and Chen et al. (2015, 2017).

In order to confirm the morphology-based identifications, representative isolates were selected from across the groups and subjected to molecular-based characterization. Genomic DNA extraction was performed on pure fungal isolates according to the in-house method (TPs 72–82 for filamentous fungi and yeasts) of the Center for Agriculture and Bioscience International (CABI), UK. Subsequently, full-length sequence of the internally transcribed spacer (ITS) 1 and 2 regions were amplified (White et al. 1990; Devarshi et al. 2013). For fungi belonging to *Aspergillus* and *Penicillium*, parts of the β-tubulin and calmodulin genes were additionally amplified (Glass and Donaldson 1995; Hong et al. 2005). Purified PCR products were Sanger sequenced at the commercial facility of CABI, UK, and the sequences obtained were matched with sequences in the European Molecular Biology Laboratory (EMBL) database through the European Bioinformatics Institute (EBI) and National Center for Biotechnology Information (NCBI) database for the identification of the fungi. The representative isolates were deposited with CABI under reference numbers E0000128001–E0000128010.

### Determination of mycotoxins in food samples

Food samples (*n* = 112: 53 melon and 59 sesame) were subjected to multi-mycotoxin analysis by the dilute and shoot LC-MS/MS method of Sulyok et al. (2020). A total of 5 g of each sample was mixed with 20 mL of extraction solvent (acetonitrile/water/acetic acid 79:20:1, v/v/v) in a 50 mL polypropylene tube (Sarstedt, Nümbrecht, Germany) and extracted for 90 min on a GFL 3017 rotary shaker (GFL, Burgwedel, Germany). The mixture was allowed to stand, and the top layer of the extracts was then diluted with the same volume of extraction solvent and injected into the LC-MS/MS instrument (Sulyok et al. 2007).

LC-MS/MS screening of the metabolites was performed with a QTrap 5500 LC-MS/MS System (Applied Biosystem, Foster City, CA, USA) equipped with TurboIonSpray electrospray ionization (ESI) source and a 1290 Series HPLC System (Agilent, Waldbronn, Germany). Chromatographic separation was performed at 25 °C on a Gemini® C18-column, 150 × 4.6 mm i.d., 5 μm particle size, equipped with a C18 4 × 3 mm i.d. security guard cartridge (Phenomenex, Torrance, CA, USA). The chromatographic method and chromatographic and mass spectrometric parameters are as documented by Sulyok et al. (2020). ESI-MS/MS was performed in the time-scheduled multiple reaction monitoring (MRM) mode both in positive and negative polarities in two separate chromatographic runs per sample by scanning two fragmentation reactions per analyte. The MRM detection window of each analyte was set to its expected retention time at ± 20 s and ± 26 s in the positive and the negative modes, respectively. Confirmation of positive analyte identification was obtained by the acquisition of two MRMs per analyte (with the exception of moniliformin (MON), which exhibited only one fragment ion). This yielded 4.0 identification points according to European Commission decision 2002/657 (EC 2002). In addition, the LC retention time and the intensity ratio of the two MRM transitions agreed with the related values of an authentic standard within 0.1 min and 30%, respectively. The accuracy of the analytical method was verified by participation in inter-laboratory comparison studies organized by BIPEA (Gennevilliers, France). At present, 94% of the over 850 results submitted for different types of foods including (grains, nuts and dried fruits) and animal feed were in the satisfactory range (z-score between − 2 and 2).

### Data analysis

Data analysis was performed on IBM Statistical Package for SPSS® 21.0 software. Student’s *t* test statistical analysis was applied to compare means of mycotoxins in the seasons (dry and wet) for each food. Microsoft Excel 2010 version was used to prepare the charts.

## Results and discussion

### Moulds associated with melon and sesame seeds

**Fig. 1:**
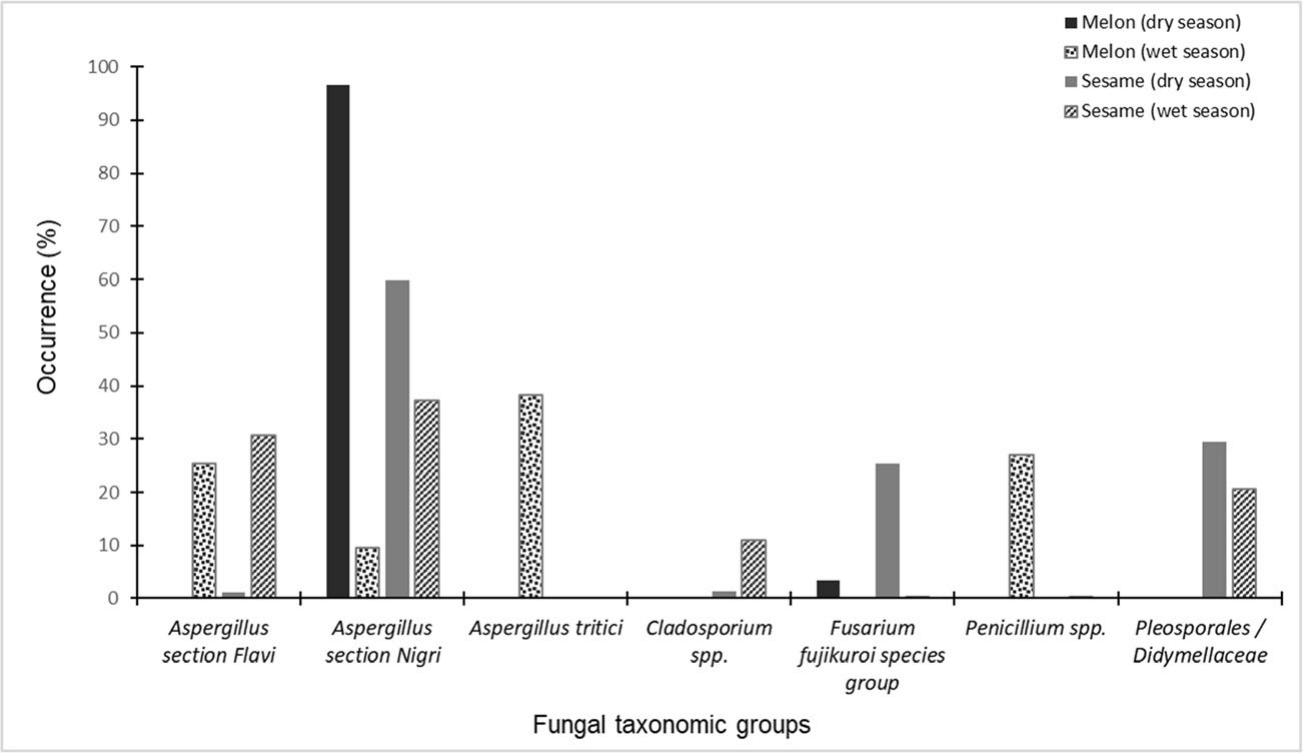
Occurrence of fungi identified in melon and sesame seeds during dry and wet seasons in Nigeria

In this study, diverse fungi, including notable storage moulds, in melon and sesame seeds marketed in Benue and Nasarawa states, respectively, were recovered. Precisely, 61 distinct fungal isolates representing seven fungal taxonomic groups (Fig. 1) were recovered from the food samples. The number of recovered fungal isolates in the present study is relatively low compared with those previously obtained from these foods in Nigeria and Senegal (Diedhiou et al. 2011; Ezekiel et al. 2014, 2016). We attribute this variation to the minimal proportion of visibly damaged (broken, discoloured and insect-infested) seeds to clean seeds in the batches of samples analysed in the present study. The overall percentage occurrences of the fungi are given as follows: *Aspergillus* section *Nigri* (39%), *Aspergillus* section *Flavi* (18%), Pleosporales/Didymellaceae (12%), *Aspergillus tritici* (representing *Aspergillus* section *Candidi*, 10%), *Fusarium fujikuroi* species group (8%), *Penicillium* spp. (8%) and *Cladosporium* spp. (5%). *Aspergillus* section *Nigri* was the dominant fungal group in both crops (melon: 44%; sesame: 35%). *Aspergillus flavus* and *A. tamarii* constituted the recovered species within *Aspergillus* section *Flavi*. The diversity of fungi and dominance of the genus *Aspergillus*, specifically species belonging to the sections *Flavi* and *Nigri*, observed in the present study agree with previous reports for fungi in diverse foods, including melon and sesame in Nigeria, suggesting these two foods harbour diverse fungal communities (Mbah and Akueshi 2000; Fagbohun et al. 2011; Adetunji et al. 2014; Ezekiel et al. 2014, 2016; Nwokocha and Opara 2016; Akoma et al. 2019). These two dominant sections of *Aspergillus* contain highly toxigenic species which are of prime importance to food safety.

Fungi belonging to all the identified taxonomic groups except *Cladosporium* and Pleosporales/Didymellaceae were recovered from the melon samples, while *A. tritici* was the only group not found in the sesame samples (Fig. 1). Overall, higher percentage occurrences of the fungi were recorded in the crops obtained during the wet season (melon: 56%; sesame: 62%) compared with the dry season (melon: 44%; sesame: 38%). Although the disparity in fungal groups recovered from the two food types may be mainly attributed to bias from the culture-dependent mycological technique adopted which favours selective isolation, as well as possible seasonal/climatic variations and influences, crop-specific colonization by the moulds cannot be ruled out. The higher colony counts of fungi during the wet season compared with the dry season may be attributed to the increased environmental humidity usually observed during the wet season, which increases viability and dissemination of fungi (Abu-Dieyeh et al. 2010; Mannaa and Kim 2017).

### Occurrence levels of mycotoxins in melon and sesame seeds

A total of 64 and 68 of microbial metabolites were found in melon (Tables 1 and S1) and sesame (Tables 1 and S2), respectively. Among the metabolites were 16 mycotoxins and other metabolites produced by several fungal genera including, but not excluded to, *Aspergillus*, *Alternaria*, *Fusarium* and *Penicillium*. The spectrum of fungal metabolites detected in the food samples agree with the diversity of moulds recovered from the foods, except for a few classes of compounds whose fungal producers were not recovered—again, owing possibly to the limitations of the applied culture-dependent mycological analysis (Ezekiel et al. 2020a).

**Table 1:** Distribution of mycotoxins in melon and sesame seeds marketed in the highest crop-producing states, Benue and Nasarawa, respectively, in Nigeria

Mycotoxins	LOD^a^ (μg kg^−1^)	Melon (*n* = 53)				Sesame (*n* = 59)				
		N (%)^b^	Range	Mean	Median	N (%)^b^	Range	Mean	Median	
Aflatoxicol	1	12 (23)	0.15–8.01	2.04	1.06	3 (5)	0.53–14.0	5.06	0.68	
Aflatoxin B_1_	0.24	40 (76)	0.14–152	9.13	1.67	7 (12)	0.29–79.3	14.8	2.67	
Aflatoxin B_2_	0.4	28 (53)	0.003–16.2	1.66	0.57	5 (8)	0.17–8.54	2.50	1.20	
Aflatoxin G_1_	0.32	13 (25)	0.17–1.68	0.52	0.32	4 (7)	0.17–0.90	0.49	0.45	
Total aflatoxins	–	40 (76)	0.14–168	10.5	2.16	7 (12)	0.29–88.5	16.9	2.84	
Aflatoxin M_1_	0.4	14 (26)	0.005–3.12	0.61	0.33	3 (5)	0.18–2.56	1.00	0.27	
Aflatoxin P_1_	0.1	0 (0)	<LOD	<LOD	<LOD	3 (5)	0.004–1.03	0.35	0.01	
Alternariol (AOH)	0.4	2 (4)	0.09–0.97	0.53	0.53	7 (12)	0.49–3.78	1.85	0.84	
AOHmethylether	0.032	5 (9)	0.28–14.5	3.72	0.62	35 (59)	0.12–47.2	4.19	0.74	
Beauvericin	0.008	5 (9)	0.19–0.71	0.34	0.23	18 (31)	0.21–42.7	3.34	0.45	
Citrinin	0.16	17 (32)	0.18–12.6	2.83	1.14	7 (12)	0.77–26.8	6.48	1.98	
Dihydrocitrinone	1.2	9 (17)	0.92–5.93	2.21	1.39	2 (3)	1.35–18.3	9.84	9.84	
Fumonisin B_1_	2	0 (0)	<LOD	<LOD	<LOD	4 (7)	5.60–24.0	13.0	11.3	
Moniliformin	1.6	0 (0)	<LOD	<LOD	<LOD	11 (19)	3.24–38.1	12.4	7.68	
Ochratoxin A	0.4	1 (2)	<LOD–112	112	112	0 (0)	<LOD	<LOD	<LOD	
Ochratoxin B	0.6	1 (2)	<LOD–94.2	94.2	94.2	0 (0)	<LOD	<LOD	<LOD	
Sterigmatocystin	0.1	34 (64)	0.03–28.1	1.71	0.44	7 (12)	0.25–11.7	3.97	0.96	

^a^Limit of detection (expressed as μg kg^−1^ sample)

^b^Number (percentage) of positive samples

Apparent recoveries were previously reported in Ezekiel et al. (2012, 2016)

In this study, a major objective was to determine the levels of mycotoxins in melon and sesame seeds sold in markets situated in the states that rank as the top crop producers in Nigeria. Thus, mycotoxins quantified in the food samples were aflatoxins, alternariol, beauvericin, citrinin, fumonisins, ochratoxins and sterigmatocystin (Table 1). Several of the aforementioned mycotoxins were previously reported in the two food types (albeit at varying concentrations) collected from different parts of Nigeria, but not in melon from Benue state (Ezekiel et al. 2012, 2016; Fapohunda et al. 2012, 2018; Somorin et al. 2016; Ogara et al. under review). Thus, we present to the best of our knowledge the first report on mycotoxin contamination data of melon in Benue state, and a comparison of toxin levels in both crops across two seasons—this is of importance due to the crop-export revenue contribution of both states to the country.

Specifically, the melon and sesame samples contained 13 and 14 mycotoxins, respectively (Table 1). Aflatoxin B_1_ was the most frequently found mycotoxin in melon seeds (occurrence: 76%) whereas alternariol monomethyl ether was predominant in the sesame seeds (occurrence: 59%). Aflatoxins quantified in the food types included B_1_, B_2_, G_1_, M_1_ and P_1_; thus, total aflatoxins were the sum of aflatoxins B_1_, B_2_ and G_1_. More samples of melon seeds (occurrence: 76%) contained aflatoxins compared with sesame seeds (occurrence: 12%); however, mean contamination levels in sesame (aflatoxin B_1_: 15 μg kg^−1^; total aflatoxins: 17 μg kg^−1^) were higher albeit not statistically significant (*p* > 0.05) compared with melon (aflatoxin B_1_: 9 μg kg^−1^; total aflatoxins: 11 μg kg^−1^). The prevalence and contamination levels of aflatoxins (aflatoxin B_1_ and total aflatoxins) in the melon seeds agree with previous reports of more than 50% prevalence and mean levels of < 15 μg kg^−1^ aflatoxin B_1_ or total aflatoxin in melon sampled from Nigeria, Ireland and the UK (Bankole et al. 2004, 2006; Williams et al. 2015; Somorin et al. 2016), except for Ezekiel et al. (2016) who documented higher mean aflatoxin levels (aflatoxin B_1_: 37.5 μg kg^−1^; total aflatoxins: 48.7 μg kg^−1^) in 81% of 16 samples from local markets in Lagos state (Nigeria). For sesame, our findings of low prevalence of contaminated samples are in line with previous studies (Mbah and Akueshi 2009; Ezekiel et al. 2012; Fapohunda et al. 2012, 2018; Makun et al. 2014). However, the mean aflatoxin levels in our samples were lower than the levels (69.72 μg kg^−1^) reported in same crop collected from Niger state (Makun et al. 2014), but higher than aflatoxin levels in all other previous reports on sesame in Turkey (Yentur et al. 2006), Iran (Asadi et al. 2011), Senegal (Diedhiou et al. 2011) and Nigeria (Mbah and Akueshi 2009; Ezekiel et al. 2012; Fapohunda et al. 2012, 2018; Ogara et al. under review). The lower mean aflatoxin levels in melon compared with sesame in this study may be attributed to the pre-cleaning/winnowing step applied to the melon samples by vendors, which was not applied to the sesame seeds. Winnowing, as with physical sorting, is a step capable off pre-cleaning grains by separating light-weighted, infect-infested and broken grains from a lot (Kaushik 2015; Matumba et al. 2015; Karlovsky et al. 2016). To the best of our knowledge, aflatoxins M_1_ and P_1_, two demethylation products of aflatoxin B_1_, are reported for the first time in sesame. Previously, we had shown the presence of aflatoxin M_1_ in melon (Ezekiel et al. 2016). Although these metabolites were previously regarded as products of endogenous biotransformation of aflatoxin B_1_ by CYP450 enzyme, they are now detectable in food crops (Warth et al. 2012; Adetunji et al. 2014; Ogara et al. 2017; Oyedele et al. 2017; Ezekiel et al. 2020b); thus, suggesting possible release of and exogenous activity of the CYP enzymes by aflatoxigenic fungi during food storage.

At present, there is no regulation for aflatoxins in both crops at the local market in Nigeria; therefore, we present data on comparison with EU limits. In the EU to where both crops are frequently exported, the maximum limits for aflatoxins in the foods (oilseeds intended for direct human consumption) are lower (2 and 4 μg kg^−1^ for aflatoxin B_1_ and total aflatoxins, respectively; European Commission 2010). Therefore, 32 and 28% of melon and 7 and 5% of sesame contained levels of aflatoxin B_1_ and total aflatoxins, respectively, above the regulations. Usually, these crops are aggregated by medium-to-large scale vendors and then transported over 10 h by road to the major international borders for shipment; transportation is often under poor storage conditions which could lead to fungal proliferation and further toxin accumulation. Thus, in view of the time-lag for transportation of crops from these states to major international borders, the poorly developed food safety system in the country mediated by substandard food handling facilities and low awareness of food producers (farmers, traders and processors, Ezekiel et al. 2013; Ojuri et al. 2019), the frequency of samples with aflatoxin levels exceeding the maximum levels and the degree to which maximum levels are exceeded may rise in crop lots that find their way to the international market chain. Based on findings in the present study, prompt and strict attention are required for pre- and post-harvest handling of these crops, especially melon.

Citrinin and sterigmatocystin were also frequently quantified in 32 and 64% of the melon samples, albeit at low mean (max) concentrations (μg kg^−1^) of 2.8 (13) and 1.7 (28), respectively. In addition, both toxins were found in 12% of the sesame samples at mean concentrations of 6 and 4 μg kg^−1^, respectively, which contrasts a recent report that did not find both toxins in 35 sesame samples collected in 2013 from markets in Nasarawa state (Ogara et al. under review). The disparity in the contamination data for sesame may be attributed to climate actions (especially increased rainfall that brings about increased moisture levels) and/or improved sensitivity of the analytical method as recently, citrinin is now commonly found in foodstuffs in Nigeria (Ojuri et al. 2018; Ezekiel et al. 2020b). Similarly, low levels of both toxins were reported in melon (Somorin et al. 2016) and sesame (Ezekiel et al. 2012). The recorded higher mean CIT level in sesame compared with melon may be due to the usual practice of storing sesame seeds in bags without underlays on cold bare floor surfaces compared with melon often stored in bags with thick (approx. 1 cm) underlays. Fumonisin B_1_ (occurrence: 7%; mean: 13 μg kg^−1^) and moniliformin (occurrence: 19%; mean: 12 μg kg^−1^) were found only in the sesame samples; both toxins at similar concentrations have been reported in sesame from Abuja and recently in Nasarawa; Fapohunda et al. 2012, 2018; Ogara et al. under review) but not from the Plateau; Ezekiel et al. 2012) Nigeria. Noteworthy to mention, Abuja and Nasarawa are characterized by slightly warmer climate (above 30 °C) than the Plateau state which is known for temperate-like climate (much lower than 25 °C) due to its elevation above sea level. In addition, rainfall pattern is more frequent in Benue compared with the aforementioned states leading to optimum temperatures of around 26–29 °C. Fumonisin production by *Fusarium verticillioides* is temperature dependent and fumonisin-producing fungi, which also biosynthesize moniliformin, have been reported to produce higher levels of fumonisins in subtropical and tropical regions (Shephard et al. 1996; Marasas 2001; Reddy et al. 2009) compared with much colder regions (Logrieco et al. 2002; Miller 2008). This may be the main reason for the disparity in fumonisin and moniliformin occurrences across the states in Nigeria.

Ochratoxins A and B were found in only one melon sample at concentrations of 112 and 94 μg kg^−1^; the ochratoxin A level reported in the present study is about 180-fold higher than the levels reported by Ezekiel et al. (2016) and Somorin et al. (2016) who found 0.6 μg kg^−1^ ochratoxin A in 6% (1 our 16) and 14% (3 out of 22) of melon samples, respectively. In the present study, however, we did not find ochratoxin A in the sesame samples. This agrees with our previous report on sesame from the Plateau state but negates the report of Makun et al. (2013) who found this toxin (range: 1.9–15.7 μg kg^−1^; mean: 8.1 μg kg^−1^) in all 19 samples analysed. Overall, grain size, natural inhibitory compounds (e.g. sesamin) and possibly seed varietal composition may have contributed to the disparity in mycotoxin levels in sesame. Makun et al. (2013) and Ezekiel et al. (2014) had suggested the two earlier points as contributory factors for low mycotoxin levels in sesame seeds. We, however, postulate that varietal composition/differences play a role in susceptibility to mycotoxin accumulation in sesame. Thus, sesame varieties in Nigeria deserve thorough examination in this regard in order to determine their susceptibilities to both toxigenic fungi and toxic metabolites. This is the first report of ochratoxin B in melon.

**Fig. 2:**
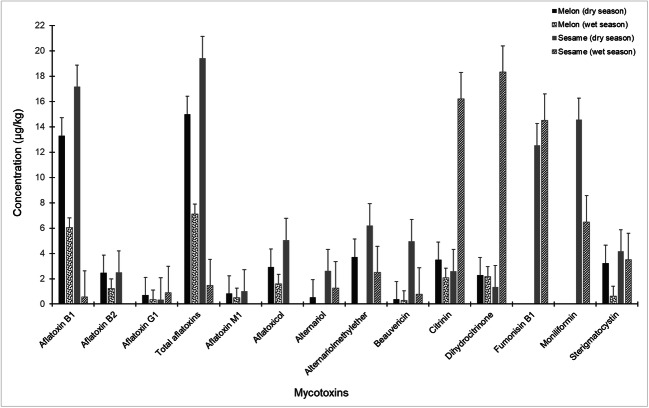
Mean mycotoxin levels in melon and sesame seeds sampled during dry and wet seasons in Nigeria. Whiskers on bars indicate standard error of means

Comparing the toxin data in each food type across the seasons (Fig. 2), higher but statistically non-significant (*p* > 0.05) mean levels of all the mycotoxins, except citrinin, dihydrocitrinone and fumonisin B_1_, were found in sesame samples from dry season compared with those from the wet season. For example, total aflatoxin levels in dry season samples (melon: 15 μg kg^−1^; sesame: 19 μg kg^−1^) were at least twice higher than levels in samples from the wet season (melon: 7 μg kg^−1^; sesame: 1.5 μg kg^−1^). The findings of our study agree well with previous reports on higher aflatoxin levels in foods collected in the dry season than in those from the wet season in Nigeria (Ojuri et al. 2018). However, the levels of citrinin, dihydrocitrinone and fumonisin B_1_ in sesame were significantly (*p* < 0.05) higher in samples from the wet season than in the dry season samples. Similarly, higher levels of citrinin and fumonisins have been reported in foods from the wet season than in those from dry seasons (Ojuri et al. 2019); and increased frequency of rainfall influences higher toxin production in some fungi (Marin et al. 1995; Ono et al. 1999). Obviously, climatic seasons influence mycotoxin production and levels in foods in Nigeria.

Overall, this study is of high relevance considering the toxicological data available in the literature on the effects of consuming aflatoxin-contaminated foods especially in high-risk regions where access to food diversity is low (IARC 2015). Furthermore, concerns of possible combinatory toxicological effects from mycotoxin mixtures, as have been previously documented (Alassane-Kpembi et al. 2016; Vejdovszky et al. 2016a, 2016b), suggest that interventions are urgent. Considering climate change, more studies focused on seasonal variations vis-à-vis mycotoxin contamination of crops/food are required in the country in order to understand the contamination trends, map hotspots and deploy targeted mitigation strategies for enhanced food security and public health.

## Electronic supplementary material

Supplementary Table captions.

Table S1. Distribution of other 51 microbial metabolites in melon seeds marketed in Nigeria.

Table S2. Distribution of other 54 microbial metabolites in sesame marketed in Nigeria.

ESM 1 (DOCX 534 kb)
