# Supplementary material for: Distribution of fungi and their toxic metabolites in melon and sesame seeds marketed in two major producing states in Nigeria
Source: Mycotoxin Res. 2020 Jul 14;36(4):361–9. doi: 10.1007/s12550-020-00400-0 (PMC7536151; doi:10.1007/s12550-020-00400-0)
Supplement: Supplementary file 1 — (DOCX 534 kb) [file 12550_2020_400_MOESM1_ESM.docx]

**Distribution of fungi and their toxic metabolites in melon and sesame seeds marketed in two major producing states in Nigeria**

**Running title: Fungi and mycotoxins in melon and sesame seeds**

**Journal:** Mycotoxin Research

**Adetoun O. Esan^1^, Stephen O. Fapohunda^1^, Chibundu N. Ezekiel^1,2,*^, Michael Sulyok^2^ and Rudolf Krska^2,3^**

^1^Department of Microbiology, Babcock University, Ilishan Remo, Ogun State, Nigeria. ^2^Institute of Bioanalytics and Agro–Metabolomics, Department of Agrobiotechnology (IFA–Tulln), University of Natural Resources and Life Sciences Vienna (BOKU), Konrad Lorenzstr. 20, A–3430 Tulln, Austria. ^3^Institute for Global Food Security, School of Biological Sciences, Queen’s University Belfast, University Road, Belfast, BT7 1NN, Northern Ireland, United Kingdom.

*Corresponding author e-mail: [chaugez@gmail.com](mailto:chaugez@gmail.com)

Table S1 Distribution of other 51 microbial metabolites in melon seeds marketed in Nigeria.

| Metabolites | LOD^a^  (µg kg^-1^) | Melon (n=53) | | | | | |
| --- | --- | --- | --- | --- | --- | --- | --- |
|  |  | N^b^ | %^c^ | Min | Max | Mean | Median |
| 3-Nitropropionic acid | 0.8 | 9 | 17.0 | 12.6 | 700 | 159 | 78.5 |
| 7-Hydroxypestalotin | 0.4 | 2 | 3.77 | 1.09 | 22.2 | 11.7 | 11.7 |
| Andrastin A | 0.25 | 2 | 3.77 | 0.06 | 1.01 | 0.53 | 0.53 |
| Aspergillicin derivative | 0.5 | 1 | 1.89 | 11.9 | 11.9 | 11.9 | 11.9 |
| Aspergillimide | 0.03 | 7 | 13.2 | 0.16 | 4.03 | 1.10 | 0.45 |
| Asperglaucide | 0.08 | 53 | 100 | 3.61 | 687 | 154 | 118 |
| Asperphenamate | 0.04 | 53 | 100 | 1.78 | 194 | 23.6 | 13.8 |
| Averantin | 0.04 | 9 | 17.0 | 0.03 | 0.96 | 0.40 | 0.29 |
| Averufin | 0.04 | 29 | 54.7 | 0.12 | 14.2 | 1.52 | 0.56 |
| Bikaverin | 8 | 26 | 49.1 | 1.48 | 28.9 | 7.46 | 5.01 |
| Brevianamid F | 0.1 | 45 | 84.9 | 0.27 | 3.31 | 1.39 | 1.27 |
| Chanoclavin | 0.08 | 1 | 1.89 | 1.42 | 1.42 | 1.42 | 1.42 |
| Chloramphenicol | 0.03 | 25 | 47.2 | 0.01 | 0.58 | 0.29 | 0.31 |
| Chlorocitreorosein | 2 | 5 | 9.43 | 0.58 | 7.35 | 3.51 | 1.87 |
| Citreorosein | 0.64 | 22 | 41.5 | 0.79 | 7.32 | 2.13 | 1.50 |
| cyclo(L-Pro-L-Tyr) | 0.8 | 47 | 88.7 | 1.64 | 25.7 | 7.47 | 6.35 |
| cyclo(L-Pro-L-Val) | 0.64 | 52 | 98.1 | 0.35 | 49.1 | 10.6 | 8.50 |
| Cyclosporin A | 4 | 8 | 15.1 | 1.11 | 42.8 | 24.2 | 20.5 |
| Cyclosporin H | 0.4 | 36 | 67.9 | 1.43 | 38.2 | 6.25 | 3.26 |
| Dihydroxymellein | 0.5 | 2 | 3.77 | 1.34 | 5.47 | 3.41 | 3.41 |
| Emodin | 0.056 | 26 | 49.1 | 0.21 | 1.20 | 0.49 | 0.40 |
| Endocrocin | 5 | 26 | 49.1 | 3.14 | 37.2 | 12.6 | 8.68 |
| Epiequisetin | 0.24 | 7 | 13.2 | 0.05 | 0.79 | 0.35 | 0.32 |
| Equisetin | 0.24 | 9 | 17.0 | 0.38 | 2.93 | 1.00 | 0.81 |
| Fallacinol | 0.1 | 43 | 81.1 | 0.08 | 20.8 | 4.37 | 2.97 |
| Flavoglaucin | 0.24 | 46 | 86.8 | 0.50 | 1470 | 189 | 40.0 |
| Iso-Rhodoptilometrin | 0.64 | 10 | 18.9 | 0.03 | 0.74 | 0.17 | 0.07 |
| Kojic acid | 16 | 36 | 67.9 | 7.97 | 1190 | 155 | 46.2 |
| Macrosporin | 0.04 | 2 | 3.77 | 0.43 | 0.89 | 0.66 | 0.66 |
| Malformin C | 0.4 | 3 | 5.66 | 0.13 | 44.9 | 15.2 | 0.56 |
| Monactin | 0.1 | 4 | 7.55 | 0.05 | 0.37 | 0.13 | 0.05 |
| Monocerin | 0.4 | 1 | 1.89 | 0.04 | 0.04 | 0.04 | 0.04 |
| N-Benzoyl-Phenylalanine | 0.064 | 53 | 100 | 0.36 | 219 | 18.4 | 10.3 |
| Neoechinulin A | 0.8 | 45 | 84.9 | 3.59 | 564 | 96.6 | 50.6 |
| Nidurufin | 0.16 | 9 | 17.0 | 0.12 | 2.33 | 0.60 | 0.21 |
| Nonactin | 0.1 | 3 | 5.66 | 0.02 | 0.09 | 0.06 | 0.09 |
| Norsolorinic acid | 0.8 | 6 | 11.3 | 0.59 | 2.47 | 0.96 | 0.67 |
| O-Methylsterigmatocystin | 0.12 | 23 | 43.4 | 0.10 | 3.40 | 0.52 | 0.28 |
| Oxaline | 0.4 | 4 | 7.55 | 0.27 | 5.62 | 2.25 | 1.55 |

Table S1. Continued.

| Metabolites | LOD^a^  (µg kg^-1^) | Melon (n=53) | | | | | |
| --- | --- | --- | --- | --- | --- | --- | --- |
|  |  | N^b^ | %^c^ | Min | Max | Mean | Median |
| Pestalotin | 0.4 | 1 | 1.89 | 26.5 | 26.5 | 26.5 | 26.5 |
| Phenopyrrozin | 1.5 | 4 | 7.55 | 0.55 | 1.51 | 1.18 | 1.33 |
| Pinselin | 0.5 | 19 | 35.9 | 0.45 | 17.0 | 2.85 | 1.55 |
| Questiomycin A | 2 | 3 | 5.66 | 2.11 | 19.3 | 8.18 | 3.16 |
| Quinolactacin A | 0.08 | 39 | 73.6 | 0.00 | 2.40 | 0.23 | 0.07 |
| Radicicol | 0.4 | 1 | 1.89 | 5.68 | 5.68 | 5.68 | 5.68 |
| Rugulusovin | 0.2 | 37 | 69.8 | 0.00 | 6.24 | 2.50 | 2.39 |
| Secalonic acid D | 4 | 3 | 5.66 | 1.26 | 39.5 | 20.7 | 21.3 |
| Sydonic acid | 0.15 | 1 | 1.89 | 1.07 | 1.07 | 1.07 | 1.07 |
| Tryptophol | 8 | 53 | 100 | 10.6 | 85.3 | 32.7 | 27.3 |
| Versicolorin A | 0.24 | 9 | 17.0 | 0.10 | 1.96 | 0.55 | 0.25 |
| Versicolorin C | 0.24 | 7 | 13.2 | 0.97 | 24.0 | 5.44 | 3.00 |

^a^Limit of detection [expressed as µg kg^-1^ sample].

^b^Number of positive samples.

^c^Percentage of positive samples.

Apparent recoveries were previously reported in Ezekiel et al. (2016).

Table S2 Distribution of other 54 microbial metabolites in sesame marketed in Nigeria.

| Metabolites | LOD^a^  (µg kg^-1^) | Sesame (n=59) | | | | | |
| --- | --- | --- | --- | --- | --- | --- | --- |
|  |  | N^b^ | %^c^ | Min | Max | Mean | Median |
| 3-Nitropropionic acid | 0.8 | 47 | 79.7 | 1.23 | 201 | 21.1 | 9.23 |
| 7-Hydroxykaurenolide | 1 | 1 | 1.69 | 3.28 | 3.28 | 3.28 | 3.28 |
| 7-Hydroxypestalotin | 0.4 | 1 | 1.69 | 9.59 | 9.59 | 9.59 | 9.59 |
| Andrastin A | 0.25 | 1 | 1.69 | 0.35 | 0.35 | 0.35 | 0.35 |
| Aspercolorin | 0.8 | 1 | 1.69 | 15.2 | 15.2 | 15.2 | 15.2 |
| Aspergillimide | 0.03 | 36 | 61.0 | 0.06 | 11.0 | 1.96 | 0.93 |
| Asperglaucide | 0.08 | 59 | 100 | 1.15 | 1830 | 117 | 12.4 |
| Asperphenamate | 0.04 | 59 | 100 | 0.57 | 145 | 17.1 | 4.75 |
| Averantin | 0.04 | 5 | 8.47 | 0.17 | 1.13 | 0.65 | 0.61 |
| Averufin | 0.04 | 17 | 28.8 | 0.14 | 6.46 | 1.20 | 0.53 |
| Barceloneic acid | 1 | 3 | 5.08 | 4.57 | 47.1 | 22.1 | 14.5 |
| Bassianolide | 0.05 | 1 | 1.69 | 0.28 | 0.28 | 0.28 | 0.28 |
| Bikaverin | 8 | 9 | 15.3 | 3.70 | 86.6 | 18.2 | 10.5 |
| Brevianamid F | 0.1 | 7 | 11.9 | 0.08 | 1.74 | 1.16 | 1.33 |
| Chanoclavin | 0.08 | 1 | 1.69 | 0.19 | 0.19 | 0.19 | 0.19 |
| Chloramphenicol | 0.03 | 6 | 10.2 | 0.10 | 0.16 | 0.12 | 0.12 |
| Chlorocitreorosein | 2 | 4 | 6.78 | 0.87 | 429 | 110 | 5.28 |
| Citreorosein | 0.64 | 16 | 27.1 | 0.55 | 85.1 | 6.83 | 1.53 |
| cyclo(L-Pro-L-Tyr) | 0.8 | 24 | 40.7 | 0.69 | 16.9 | 2.81 | 1.39 |
| cyclo(L-Pro-L-Val) | 0.64 | 58 | 98.3 | 1.66 | 28.4 | 5.98 | 5.20 |
| Cytochalasin D | 0.25 | 3 | 5.08 | 1.67 | 11.1 | 4.85 | 1.75 |
| Deoxyfusapyron | 0.8 | 4 | 6.78 | 1.52 | 9.58 | 5.61 | 5.68 |
| Emodin | 0.056 | 52 | 88.1 | 0.15 | 22.3 | 0.97 | 0.45 |
| Endocrocin | 5 | 6 | 10.2 | 6.03 | 448 | 85.2 | 13.8 |
| Epiequisetin | 0.24 | 8 | 13.6 | 0.01 | 3.31 | 0.71 | 0.12 |
| Equisetin | 0.24 | 10 | 17.0 | 1.17 | 6.88 | 3.84 | 3.34 |
| Fallacinol | 0.1 | 5 | 8.47 | 2.83 | 14.1 | 6.22 | 3.85 |
| Flavoglaucin | 0.24 | 17 | 28.8 | 0.34 | 224.6 | 44.2 | 3.88 |
| Hydroxysydonic acid | 1.6 | 1 | 1.69 | 111 | 111 | 111 | 111 |
| Iso-Rhodoptilometrin | 0.64 | 13 | 22.0 | 0.04 | 2.23 | 0.28 | 0.10 |
| Kojic acid | 16 | 11 | 18.6 | 18.4 | 311 | 88.8 | 38.3 |
| Macrosporin | 0.04 | 10 | 17.0 | 0.25 | 5.14 | 1.30 | 0.46 |
| Monocerin | 0.4 | 5 | 8.47 | 0.37 | 5.13 | 2.25 | 1.78 |
| N-Benzoyl-Phenylalanine | 0.064 | 54 | 91.5 | 0.33 | 62.9 | 4.42 | 1.39 |
| Neoechinulin A | 0.8 | 12 | 20.3 | 1.02 | 89.0 | 27.5 | 10.3 |
| Nidurufin | 0.16 | 5 | 8.47 | 0.30 | 4.83 | 2.04 | 1.23 |
| Norsolorinic acid | 0.8 | 5 | 8.47 | 0.79 | 3.17 | 1.91 | 2.01 |
| O-Methylsterigmatocystin | 0.12 | 7 | 11.9 | 0.11 | 1.33 | 0.67 | 0.81 |
| Oxaline | 0.4 | 38 | 64.4 | 0.02 | 11.5 | 1.71 | 0.83 |

Table S2. Continued.

| Metabolites | LOD^a^  (µg kg^-1^) | Sesame (n=59) | | | | | |
| --- | --- | --- | --- | --- | --- | --- | --- |
|  |  | N^b^ | %^c^ | Min | Max | Mean | Median |
| Pestalotin | 0.4 | 2 | 3.39 | 4.64 | 6.42 | 5.53 | 5.53 |
| Phenopyrrozin | 1.5 | 3 | 5.08 | 2.21 | 4.00 | 3.19 | 3.37 |
| Pinselin | 0.5 | 4 | 6.78 | 2.86 | 26.6 | 10.3 | 5.83 |
| Purpactin A | 0.7 | 1 | 1.69 | 0.98 | 0.98 | 0.98 | 0.98 |
| Questiomycin A | 2 | 2 | 3.39 | 6.66 | 12.2 | 9.44 | 9.44 |
| Quinolactacin A | 0.08 | 15 | 25.4 | 0.03 | 3.13 | 0.30 | 0.09 |
| Rugulovasine A | 1.7 | 1 | 1.69 | 6.33 | 6.33 | 6.33 | 6.33 |
| Rugulusovin | 0.2 | 7 | 11.9 | 1.11 | 2.76 | 2.24 | 2.30 |
| Secalonic acid D | 4 | 8 | 13.6 | 0.53 | 35.6 | 9.99 | 6.18 |
| seco-Sterigmatocystin | 0.15 | 2 | 3.39 | 0.21 | 4.98 | 2.59 | 2.59 |
| Skyrin | 0.15 | 1 | 1.69 | 1.96 | 1.96 | 1.96 | 1.96 |
| Sydonic acid | 0.15 | 1 | 1.69 | 11.8 | 11.8 | 11.8 | 11.8 |
| Tryptophol | 8 | 59 | 100 | 8.72 | 32.9 | 19.2 | 17.9 |
| Versicolorin A | 0.24 | 2 | 3.39 | 1.81 | 2.89 | 2.35 | 2.35 |
| Versicolorin C | 0.24 | 6 | 10.2 | 1.00 | 13.5 | 4.89 | 3.18 |

^a^Limit of detection [expressed as µg kg^-1^ sample].

^b^Number of positive samples.

^c^Percentage of positive samples.

Apparent recoveries were previously reported in Ezekiel et al. (2012).
